# Editorial: Dry Electroencephalography for Brain Monitoring in Sports and Movement Science

**DOI:** 10.3389/fnins.2021.809227

**Published:** 2021-12-02

**Authors:** Silvia Comani, Maurizio Bertollo, Jens Haueisen

**Affiliations:** ^1^Behavioral Imaging and Neural Dynamics Center, University “G. d'Annunzio” of Chieti–Pescara, Chieti, Italy; ^2^Department of Neurosciences, Imaging and Clinical Sciences, University “G. d'Annunzio” of Chieti–Pescara, Chieti, Italy; ^3^Department of Medicine and Aging Sciences, University “G. d'Annunzio” of Chieti–Pescara, Chieti, Italy; ^4^Institute of Biomedical Engineering and Informatics, Technische Universität Ilmenau, Ilmenau, Germany; ^5^Department of Neurology, Biomagnetic Center, Jena University Hospital, Jena, Germany

**Keywords:** EEG, dry electrode, sports science, artifact removal methods, wireless EEG

Electroencephalography (EEG) is one of the few neuroimaging methods with excellent time-resolution required to measure rapid brain signal changes and that is also lightweight enough to be worn by a moving subject. EEG monitoring in sports science and other mobile applications is thus a new area of research that provides new insights e.g., into the nature of athlete performance, offering an effective means for neurofeedback training and EEG feature control to improve performance.

The increased use of EEG in sports science applications has been aided by the recent advances in EEG data acquisition, hardware portability, and reduced preparation times, although mobile EEG systems are still affected by several limitations, the most relevant for sports applications being that signal quality is subject to sweat and motion effects. Dry electrode technology, with its unique characteristics of fast electrode preparation, no risks of short-circuits between adjacent electrodes, and long-term recording ability with no signal degradation due to sweat, is admittedly the most suitable one for movement science applications.

While these enhancements have made possible the practical application of EEG in sports science, commercial systems mounting dry electrodes are still affected by the generally low number of electrodes and low sampling frequency, preventing advanced analysis of brain activity, and by low subject comfort due to the electrode shape and material. Also, the lack of conductive gel translates into a much higher electrode/scalp impedance and a higher sensitivity to motion artifacts. Therefore, acquiring informative EEG data from participants actively engaged in physical activity continues to present many challenges and deserves the attention of the scientific community.

This article collection describes recent advancements in dry electrode technology for sport and movement science applications, and the newest biosignal processing and classification solutions for dealing with artifacts affecting EEG signals collected during motion. Dry electrode technology, with its short preparation time, miniaturization of hardware components, and wireless solutions, permits an ecological EEG data collection to investigate the cognitive and affective processes underlying performance during practice and competition in real settings.

Vasconcelos et al. present “The Arch Electrode: A Novel Dry Electrode Concept for Improved Wearing Comfort.” Their electrode includes five semi-circular flexible arches arranged parallelly on a common baseplate. It is coated with Silver/Silver-Chloride using an electroless plating and a novel surface functionalization method. The signal quality of the Arch electrodes was comparable to the established pin-shaped dry electrodes (Fiedler et al., 2015) while the comfort was improved.

The article “Automatic Removal of Cardiac Interference (ARCI): A New Approach for EEG Data” by Tamburro et al. focuses on the development of an ICA-based automated method for removing cardiac interference from EEG signals acquired with both wet and dry electrodes. The assessment of the method performance led to an overall accuracy >99% in all datasets and signal decomposition levels, and an average sensitivity >90%. Best results were attained when EEG signals were decomposed into a fewer number of components where the method achieved perfect sensitivity (100%).

The article “Dry EEG in Sports Sciences: A Fast and Reliable Tool to Assess Individual Alpha Peak Frequency Changes Induced by Physical Effort” by di Fronso et al. describes the results of a counterbalanced repeated-measure study on endurance cycling designed to test the performance of a 64-channel dry electrode cap and to verify or reject the hypothesis of a systematic, reproducible individual Alpha peak frequency (iAPF) shift consequent to an exhaustive physical task. Results showed that the average channel reliability of the dry cap was only slightly lower than that of the gel-based cap, that comfort and signal characteristics were comparable in the dry and wet caps and that the electrode type did not affect the iAPF evaluation. The authors concluded that these findings confirm the usefulness of dry electrodes in sports science and other mobile applications involving ample movements.

In their article “The Impact of Vigorous Cycling Exercise on Visual Attention: A Study With the BR8 Wireless Dry EEG System,” Lin et al. explore the impact of exercise at varying intensities on visual attention using a BR8 wireless dry-sensor EEG system to assess the potential of this system for practical application in sports. The authors showed that preparation time to attain the required skin-sensor interface impedance was generally <2 min, that different P300 amplitudes for the target and non-target responses were observed, with decreased reaction times to the visual attention task during vigorous exercise. The authors concluded that these results demonstrate the quality and reliability of EEG measurement using dry electrodes and that wireless dry EEG devices can open avenues for real-time measurement of cognitive functions in athletes outside the laboratory.

The opinion paper “From the Lab to the Field: Potential Applications of Dry EEG Systems to Understand the Brain-Behavior Relationship in Sports” by Wang et al. outlines the potential application of the dry EEG in the sport context. Drawing on the literature about the MoBI imaging that suggests linking cortical dynamics to motor behavior (Makeig et al., 2009) and on the current literature that highlights the benefits of using brain technologies in practice (Bertollo et al., 2020), the authors recommend EEG as a window to understanding brain dynamics of expert behavior in sports. They underline the utility of dry EEG systems in moving sports neuroscience toward real-world measurements to investigate brain-behavior relationships in sports.

Finally, Kang et al. report on a clinical trial titled “Meditative Movement Affects Working Memory Related to Neural Activity in Adolescents: A Randomized Controlled Trial” investigating how cognitive processes are affected by yoga, qigong, and tai chi, which involve conscious movement, meditative state of mind, attention to breathing, and deep relaxation. The researchers used wireless dry EEG to record brain activity during the dual n-back task and found that working memory performance significantly increased following meditative movement compared to a control group. Importantly, in the post-experimental group, they found a tightly coupled neuronal activity with working memory in the beta band at the F3 electrode site.

## Author Contributions

All authors listed have made a substantial, direct, and intellectual contribution to the work and approved it for publication.

## Funding

This work was supported by the European Union (FP7-PEOPLE-2013-IAPP, grant ID 610950; H2020-MSCA-ITN-2018, grant ID 813483), the German Academic Exchange Service (grant ID 57452734), and the German Federal Ministry for Economic Affairs and Energy (grant ID ZF4112007TS9).

## Conflict of Interest

The authors declare that the research was conducted in the absence of any commercial or financial relationships that could be construed as a potential conflict of interest.

## Publisher's Note

All claims expressed in this article are solely those of the authors and do not necessarily represent those of their affiliated organizations, or those of the publisher, the editors and the reviewers. Any product that may be evaluated in this article, or claim that may be made by its manufacturer, is not guaranteed or endorsed by the publisher.
